# Establishment of a prognostic signature based on fatty acid metabolism genes in HCC associated with hepatitis B

**DOI:** 10.1186/s12876-023-03026-5

**Published:** 2023-11-13

**Authors:** Ping Yan, Yunhai Luo, Zuotian Huang, Tong Mou, Hang Yang, Dadi Peng, Zhongjun Wu

**Affiliations:** 1 Department of Hepatobiliary Surgery, The First Affiliated Hospital of Chongqing Medical University, No. 1 Youyi Road, Yuzhong District, Chongqing, 400016 China; 2 Department of Hepatobiliary Pancreatic Tumor Center, Chongqing University Cancer Hospital, Chongqing, 400030 China

**Keywords:** Hepatocellular carcinoma, HBV, Fatty acid metabolism, Prognosis, Tregs

## Abstract

**Background:**

Hepatitis B virus (HBV)-associated hepatocellular carcinoma (HCC) is one of the most common and deadly cancer and often accompanied by varying degrees of liver damage, leading to the dysfunction of fatty acid metabolism (FAM). This study aimed to investigate the relationship between FAM and HBV-associated HCC and identify FAM biomarkers for predicting the prognosis of HBV-associated HCC.

**Methods:**

Gene Set Enrichment Analysis (GSEA) was used to analyze the difference of FAM pathway between paired tumor and adjacent normal tissue samples in 58 HBV-associated HCC patients from the Gene Expression Omnibus (GEO) database. Next, 117 HBV-associated HCC patients from The Cancer Genome Atlas (TCGA) database were analyzed to establish a prognostic signature based on 42 FAM genes. Then, the prognostic signature was validated in an external cohort consisting of 30 HBV-associated HCC patients. Finally, immune infiltration analysis was performed to evaluate the FAM-related immune cells in HBV-associated HCC.

**Results:**

As a result, FAM pathway was clearly downregulated in tumor tissue of HBV-associated HCC, and survival analysis demonstrated that 12 FAM genes were associated with the prognosis of HBV-associated HCC. Lasso-penalized Cox regression analysis identified and established a five-gene signature (ACADVL, ACAT1, ACSL3, ADH4 and ECI1), which showed effective discrimination and prediction for the prognosis of HBV-associated HCC both in the TCGA cohort and the validation cohort. Immune infiltration analysis showed that the high-risk group, identified by FAM signature, of HBV-associated HCC had a higher ratio of Tregs, which was associated with the prognosis.

**Conclusions:**

Collectively, these findings suggest that there is a strong connection between FAM and HBV-associated HCC, indicating a potential therapeutic strategy targeting FAM to block the accumulation of Tregs into the tumor microenvironment of HBV-associated HCC.

**Supplementary Information:**

The online version contains supplementary material available at 10.1186/s12876-023-03026-5.

## Background

Hepatocellular carcinoma (HCC), the most common primary liver cancer, is reported to be the sixth prevalent and the third most frequent cause of mortality worldwide [1]. In China, HCC is the third prevalent and second most deadly, and the incidence of HCC in China accounts for more than 50% of worldwide [2, 3]. Hepatitis B virus (HBV) infection is one of the main etiologic factor leading to the incidence of HCC all over the world, especially in eastern Asia and Africa [4]. With increasing viral load and duration of infection, HBV-initiated tumorigenic development often follows from or accompanies long-term chronic hepatitis, cirrhosis, and has a poor prognosis [5].

Metabolic dysregulation is a typical characteristic of cancer, and increasing evidence suggests that metabolic reprogramming plays a crucial role in oncogenesis and progression [6–8]. Tumor cells are often in an abnormal metabolic environment because of the imbalance between the rapid proliferation and nutrient angiogenesis. Accordingly, tumor cells need to reprogram their metabolism to meet increased metabolic and synthetic demands in a relatively nutrient-stressed tumor microenvironment (TME). However, metabolism disorder of tumor cells often lead to changes in the components of the TME, thereby having a significant impact on the biological process of other cellular components of the TME, and these changes will eventually affect tumor progression [9–11]. Fatty acids (FAs), as an important part of lipid metabolism, are required for many fundamental cellular biological processes. Dysregulation of fatty acids can not only interfere with the efficacy of chemotherapy and radiotherapy in cancer patients but also affect immunotherapy, which is a breakthrough in tumor therapy recently [12–14]. More and more evidence shows that fatty acid metabolism (FAM) has a certain relationship with the occurrence and development of tumors and is crucial for the maintenance of the TME [15–17]. However, the relationship between FAM dysfunction with HBV-associated HCC progression and clinical prognosis, and its effect in the induced TME, is yet unknown and requires further investigation.

In this study, most of the FAM genes were found to be significantly downregulated in tumor tissue of HBV-associated HCC. Based on FAM genes, a prognostic signature, consisting of five genes (ACADVL, ACAT1, ACSL3, ADH4 and ECI1), was established, which showed good performance for the prognosis of HBV-associated HCC both in the TCGA cohort and the validation cohort. Immune infiltration analysis demonstrated that the high-risk group of HBV-associated HCC had a higher ratio of Tregs, which exerts immunosuppressive effect in TME. These findings suggest that immunotherapies targeting FAM could be a strategy to block the accumulation of Tregs into the TME of HBV-associated HCC.

## Materials and methods

### Data collection

**Fig. 1:**
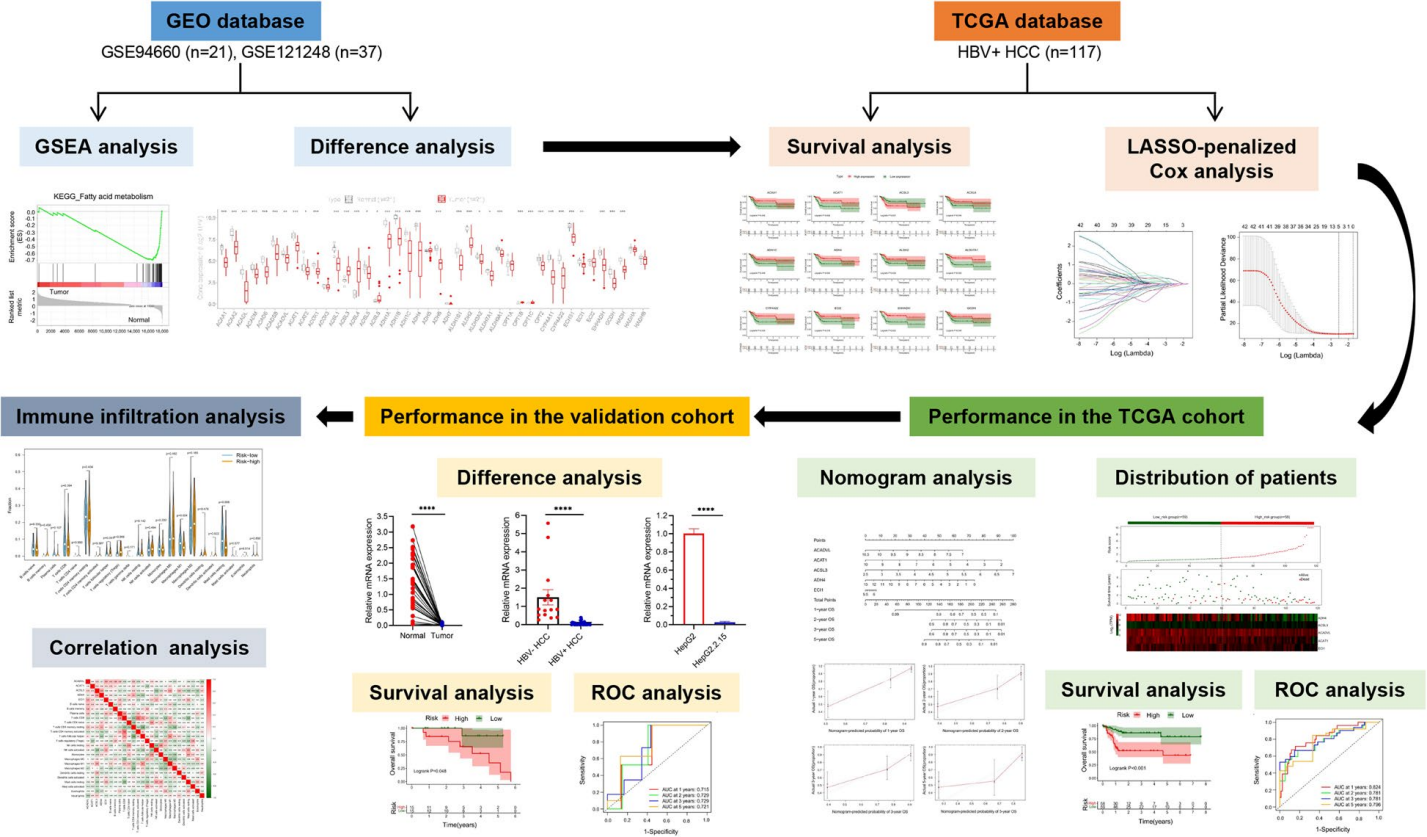
Flowchart of data collection and method implementation

Two HBV-associated HCC microarray datasets, GSE94660 [18] and GSE121248 [19], with paired tumor and adjacent normal tissue samples were obtained from the GEO database (https://www.ncbi.nlm.nih.gov/geo/). Respectively, there are 21 pairs of HBV-associated HCC samples from GSE94660 and 37 pairs from GSE121248. The RNA sequencing (RNA-seq) data of 117 HBV-associated HCC patients was downloaded from the TCGA database (https://portal.gdc.cancer.gov/). The detailed process is presented in Fig. 1.

### GSEA analysis

GSEA analysis was employed to investigate the potential signaling pathways between two groups to show possible molecular mechanisms underlying the prognosis. The Type was set to “phenotype” and the Replacement to “1000”, respectively. “c2.cp.kegg.v2022.1.Hs.symbols” was adopted to perform GSEA analysis to evaluate the Kyoto Encyclopedia of Genes and Genomes (KEGG) enrichment of Fatty Acid Metabolism pathway, provided by http://www.kegg.jp/kegg/kegg1.html.

### Survival analysis

Kaplan-Meier survival analysis with a log-rank test was performed to analyze the OS and RFS of HBV-associated HCC patients from the cohort of TCGA database and the cohort of the First Affiliated Hospital of Chongqing Medical University. *P*-value< 0.05, identified by the log-rank test, was set as statistically significant difference.

### Establishment of a prognostic signature

In the TCGA database, there were 117 HBV-associated HCC patients with overall survival time and status information, and 104 with reccurence information. Based on the identified 42 FAM genes, LASSO-penalized Cox regression analysis was performed by R package “glmnet” with 10-fold cross-validation, 1000 cycles to establish a multi-gene prognostic signature with the OS information of HBV-associated HCC patients. The risk score (RS) was calculated using the sum of the identified FAM gene expression values weighted by the coefficients from the LASSO-penalized Cox regression model. The prognostic RS for each patient could be calculated by the following formula: RS = (β1 × expression of gene1) + (β2 × expression of gene2) + ... + (βn × expression of genen).

### Performance of the prognostic signature

All the samples were categorized into a high-risk group and a low-risk group according to the median value of the RS. As described before [20], to evaluate the discrimination and prediction abilities of the RS system in both OS and RFS, the Kaplan-Meier survival analysis results were assessed in R software, time-dependent receiver operating characteristic (ROC) curve analysis was conducted, and the area under the ROC curve (AUC) was calculated in R software. Moreover, a nomogram was build to investigate the probability of 1-, 2-, 3-, and 5-OS and -RFS of HBV-associated HCC. *P*-value< 0.05 was considered to indicate statistical significance.

### Validation of the prognostic signature

The mRNA expression level of the five genes in the prognostic signature was validated in the cohort of Chongqing Medical University, consisting of 30 pairs of HBV-positive and 15 pairs of HBV-negative HCC tumor tissue and adjacent normal tissue. The RS for each HBV-positive HCC patient was calculated with the same prognostic signature identified before. Likewise, the Kaplan-Meier curve, the ROC curve and nomogram were used to evaluate the predictive performance of the prognostic gene signature on both OS and RFS.

### Immune infiltration analysis

The CIBERSORT algorithm is developed by Newman et al. [21] to estimate the abundance of member cells in a mixed cell population. Loading the R package “e1071” as a precondition, CIBERSORT algorithm was used to evaluate the relative proportion of 22 immune cells among different groups.

### Quantitative real-time PCR

As described in the previous study [20]. In brief, total RNA was extracted by TRIzol (Invitrogen, Carlsbad, CA). Then, RNA was reverse transcribed to cDNA using GoScript (Promega, Madison, WI). Finally, quantitative real-time polymerase chain reaction (qRT-PCR) was performed to analyze the gene expression level by using TB Green Premix Ex Taq (Takara, Tokyo, Japan). The expression of b-actin was set as the internal control. The primers are listed in Table 1.

**Table 1:** Primers used for quantitative real-time PCR

Gene	Forward sequences	Reverse sequences
ACADVL	5′-ACAGATCAGGTGTTCCCATACC-3′	5′-CTTGGCGGGATCGTTCACTT-3′
ACAT1	5′-GAATAGTAGCATTTGCTGACGCTG-3′	5′-AATCCTGGCTCCAGACATCCTAA-3′
ACSL3	5′-AGGAGGTCCAGCCATTGTTC-3′	5′-CTATGAGGTTGGTTTTCCATGCT-3′
ADH4	5′-CCAGGAGTGACCAACGTCAAA-3′	5′-ACCACAGTGTACTGAGAGAATGT-3′
ECI1	5′-CCTGACGGAGATGTGTGGG-3′	5′-TTGAGTCCTATGCAGTACCTGGG-3′
β-Actin	5′-CATGTACGTTGCTATCCAGGC-3′	5′-CTCCTTAATGTCACGCACGAT-3′

### Patients and samples

Paired HCC tumor tissues and adjacent normal tissues, including thirty HBV-positive HCC and fifteen HBV-negative HCC, were obtained between January 2015 and April 2016 from the First Affiliated Hospital of Chongqing Medical University. All the samples were collected with informed consent of patients and all the experiments were approved by the ethics committee of the First Affiliated Hospital of Chongqing Medical University.

### Cell culture

HepG2 and HepG2.2.15 cells were obtained from the ATCC. Cells were cultured in MEM with 10% FBS. Cell lines were confirmed to be Mycoplasma free authenticated by Short Tandem Repeat (STR) profiling.

### Statistical analysis

Kaplan-Meier survival analysis, LASSO-penalized Cox regression analysis, time-dependent receiver operating characteristic (ROC) curve analysis, nomogram analysis, correlation analysis and R software (version 4.2.1) are used. Data are presented as mean ± SEM. The gene expression differences between tumor and paired adjacent normal tissue were compared using paired Student’s t-tests. While statistical significance between HBV-positive and -negative HCC groups, and different cell groups was evaluated by unpaired Student’s t-test using GraphPad Prism software (version 8.3.0). Throughout the text, figures, and figure legends, the following terminology is used to denote statistical significance: *p < 0.05, **p < 0.01, ***p < 0.001, ns, no significance.

## Results

### GSEA analysis

**Fig. 2:**
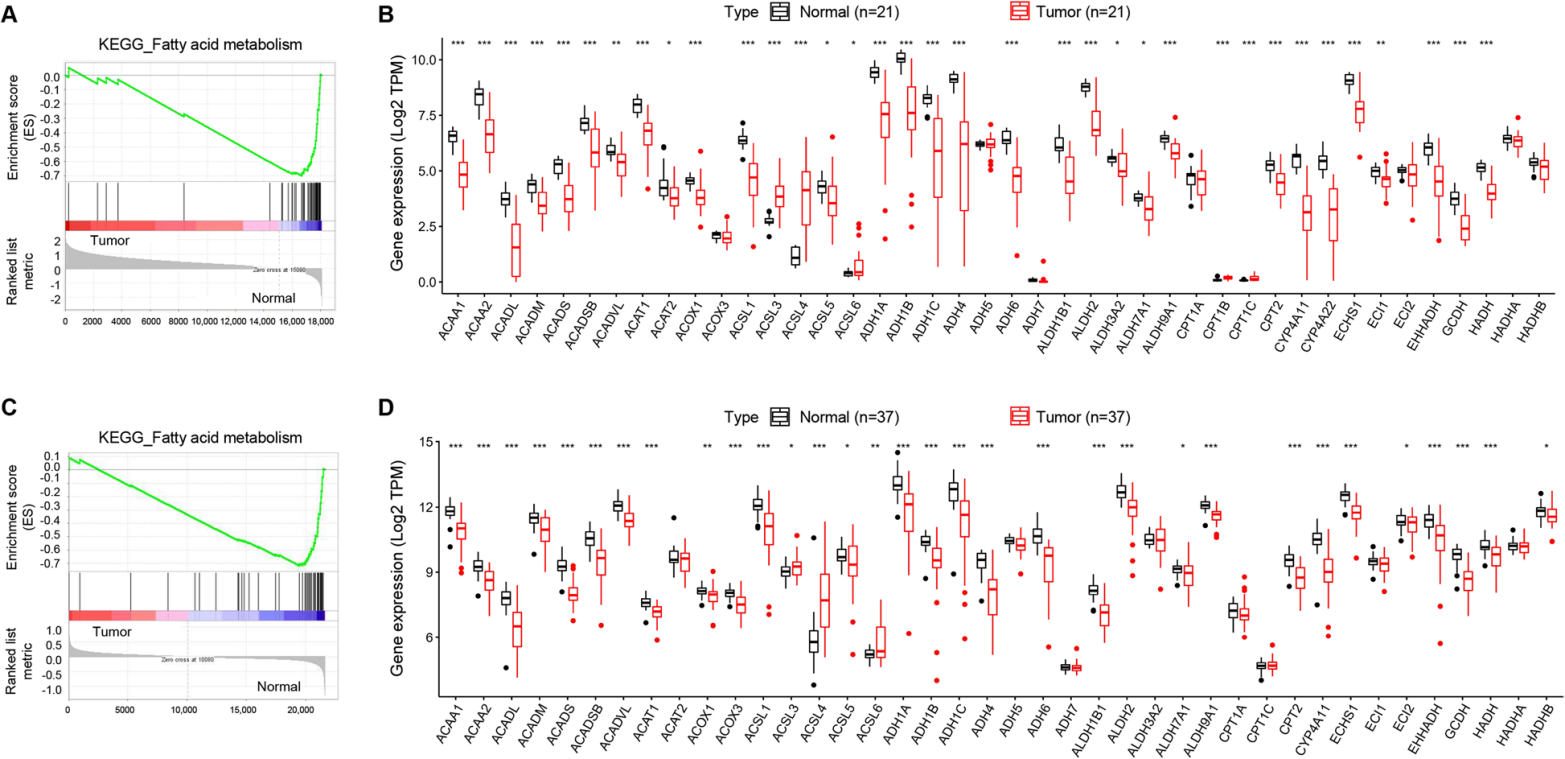
GSEA analysis of FAM pathway in HBV-associated HCC and paired normal tissue. **A** GSEA results in GSE94660; **B** Specific FAM genes in GSE94660; **C** GSEA results in GSE121248; **D** Specific FAM genes in GSE121248

To investigate the performance of Fatty Acid Metabolism pathways between tumor and adjacent normal tissue of HBV--associated HCC patients, KEGG-based GSEA analysis was performed in two GEO datasets, including GSE94660 and GSE121248. As a result, FAM pathway was clearly enriched in adjacent normal tissue both in GSE94660 (Fig. 2A) and GSE121248 (Fig. 2C). Moreover, Fig. 2B and Fig. 2D respectively showed the expression difference of 42 FAM genes between tumor and adjacent normal tissue. Moreover, we found that FAM pathway was even more down-regulated in HBV-positive HCC than in HBV-negative HCC (Fig. S1A and S1B). In a word, these results imply that FAM pathway is significantly dysregulated in HBV-associated HCC.

### Construction of the prognostic signature

**Fig. 3:**
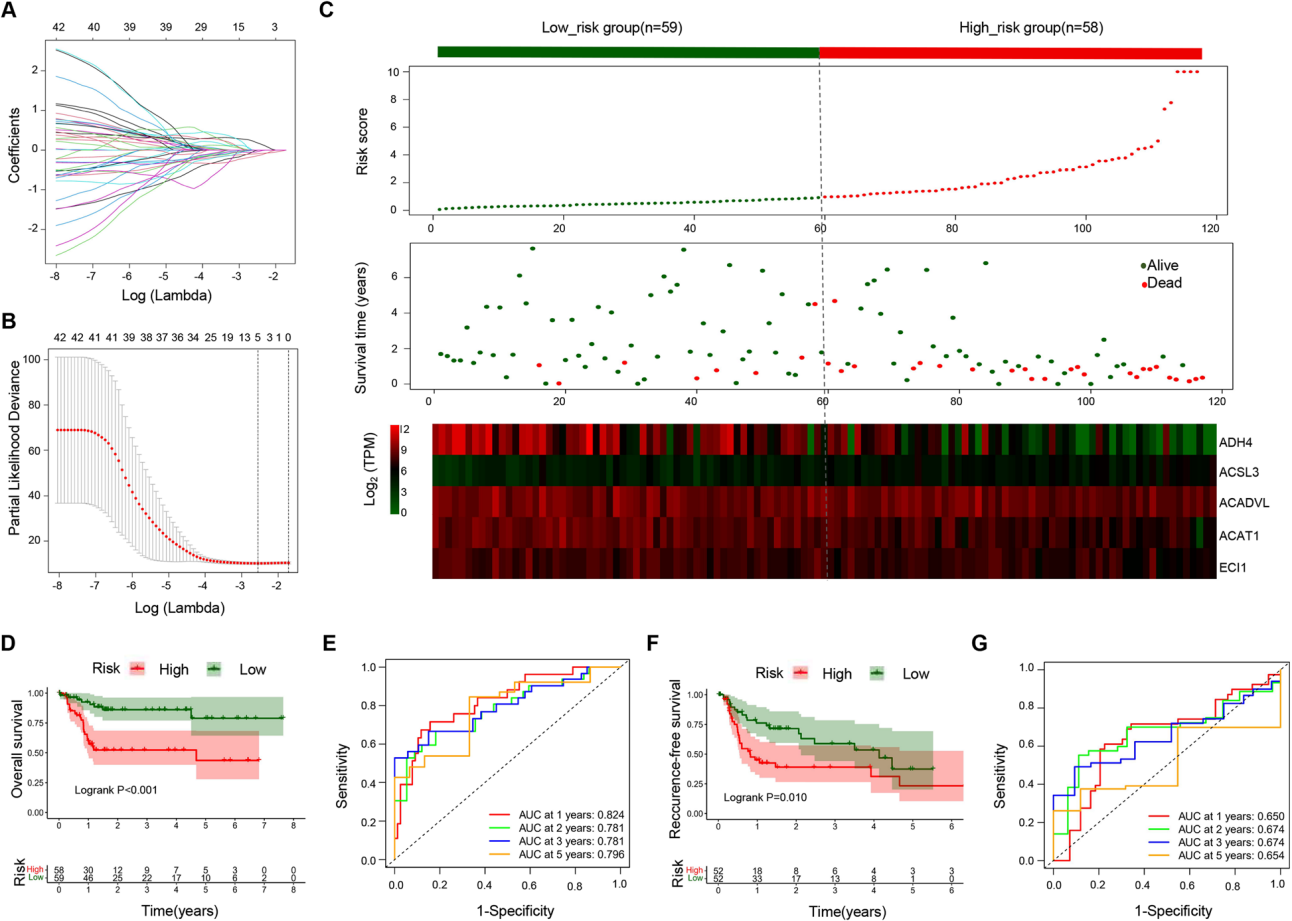
Establishment of the prognostic signature for HBV-associated HCC based on 42 FAM genes in the TCGA database. **A** LASSO regression coefficient profile of the 42 FAM genes; **B** LASSO deviance profile of the 42 FAM genes; **C** From top to bottom are the risk score distribution, survival and death status distribution, and heat map of genes in the prognostic signature between the low-risk and high-risk groups; **D** Kaplan-Meier curves for the OS; **E** Time-dependent ROC curves for predicting OS; **F** Kaplan-Meier curves for the RFS; **G** Time-dependent ROC curves for predicting RFS

With the OS information of 117 HBV-associated HCC patients in the TCGA database, Kaplan-Meier survival analysis and Lasso-penalized Cox regression analysis for 42 FAM genes was conducted. As a result, nearly 1/3 FAM genes (12/42) were associated the OS (Fig. S2), indicating that the FAM genes are significantly correlated with the prognosis of HBV-associated HCC. Moreover, a multigene signature consisting of 5 FAM genes (ACADVL, ACAT1, ACSL3, ADH4, and ECI1) was selected to predict OS in patients with HBV-associated HCC (Fig. 3A and Fig. 3B). The RS formula was as follows: RS = (− 0.5362 × expression of ACADVL) + (− 0.3284 × expression of ACAT1) + (0.6345 × expression of ACSL3) + (− 0.1621 × expression of ADH4) + (− 0.0467 × expression of ECI1). The HBV-associated HCC patients were divided into the high-risk group and the low-risk group according to the median RS. Figure 3C showed the distributions of the RS, survival status and gene expression levels between the low-risk and high-risk groups. The higher RSs the patients were, the shorter OS times and the more probability of death they might get.

### Performance in the TCGA cohort

**Fig. 4:**
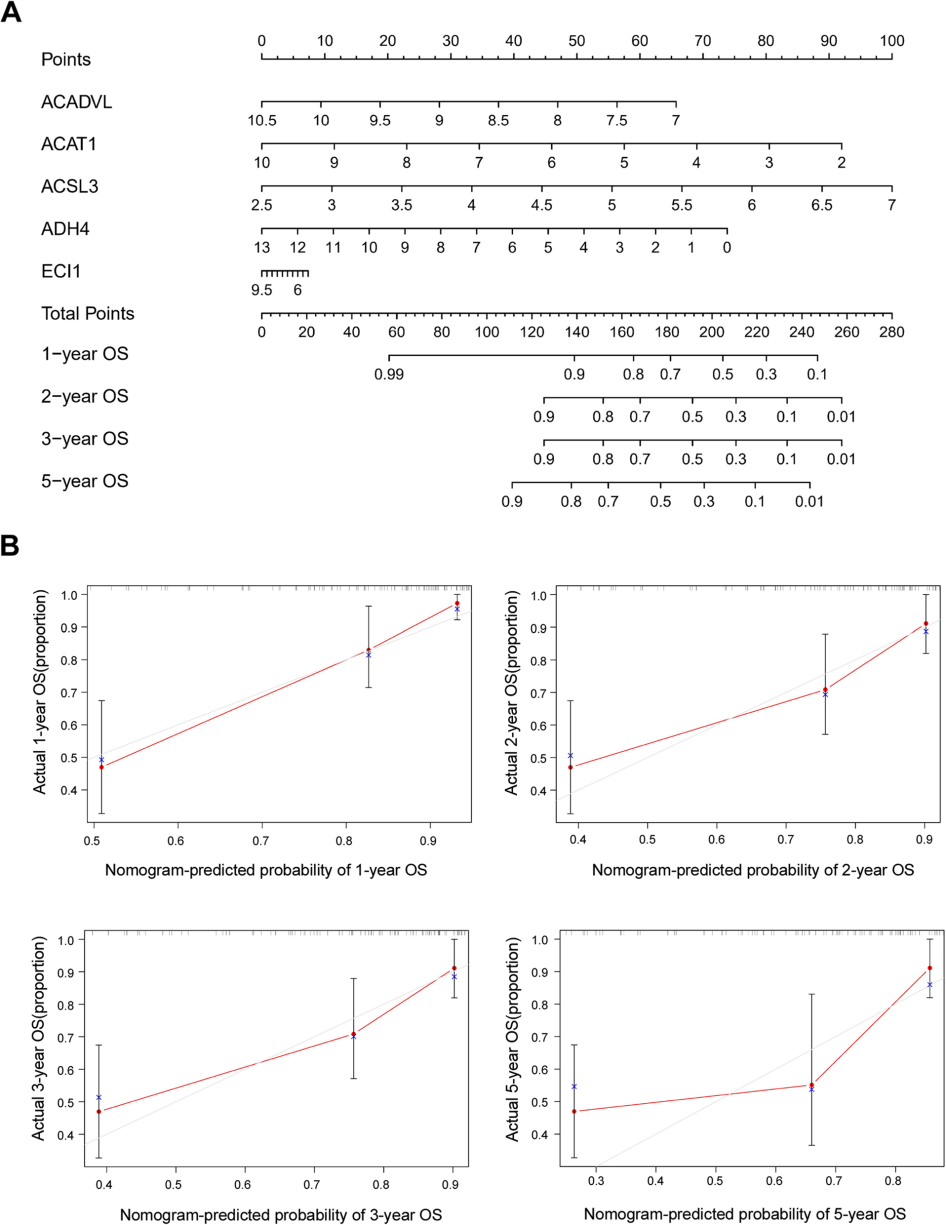
Nomogram of the prognostic signature for predicting HBV-associated HCC OS at 1-, 2-, 3-, and 5-year in the TCGA database (*n* = 117)

**Fig. 5:**
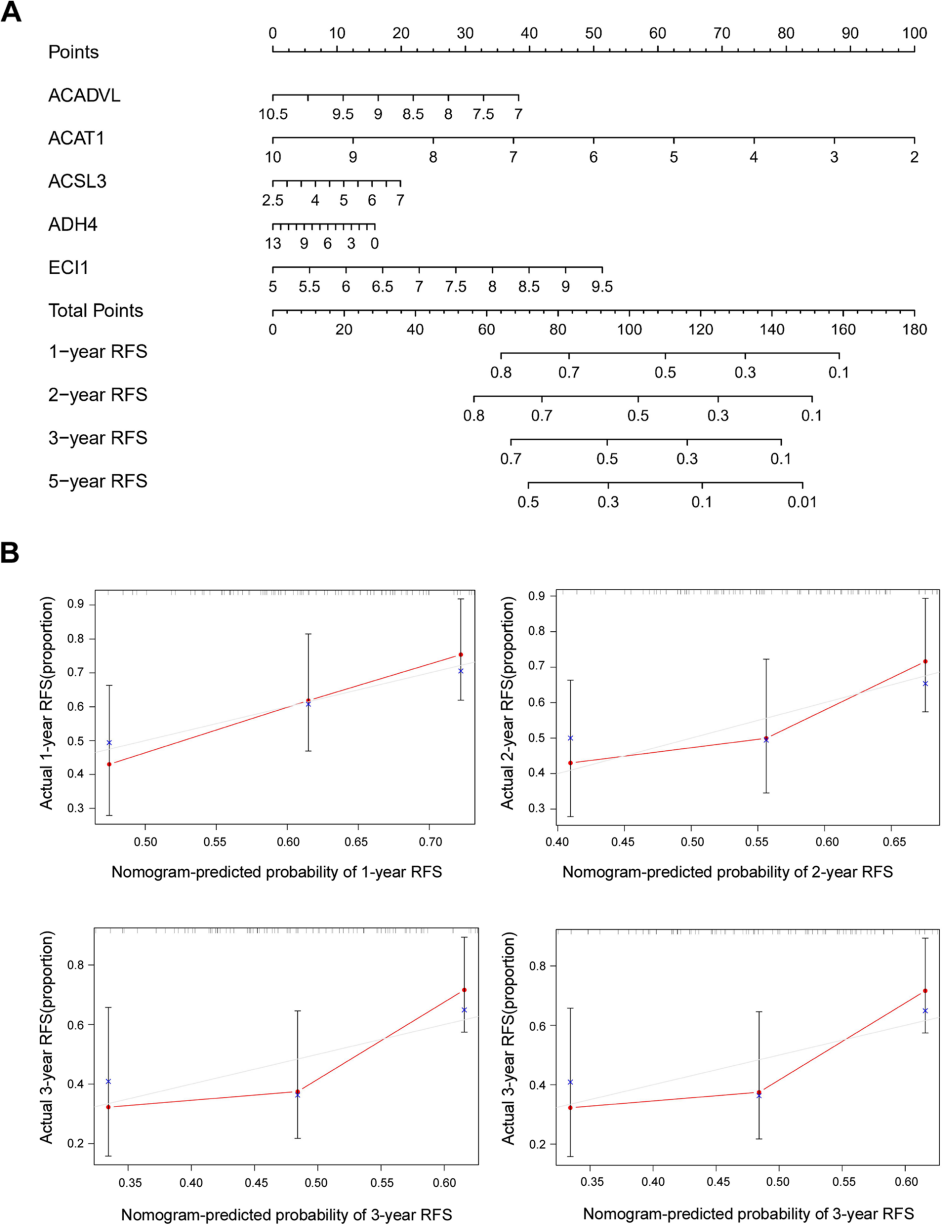
Nomogram of the prognostic signature for predicting HBV-associated HCC RFS at 1-, 2-, 3-, and 5-year in the TCGA database (*n* = 117)

In the TCGA database, 33 of 117 (28.20%) HBV-associated HCC patients died and 48 of 104 (46.15%) relapsed during the follow-up period. Kaplan-Meier analysis indicated that patients in the high-risk group had shorter OS and RFS time than those in the low-risk group (*P*-value< 0.05) and were more likely to experience death (Fig. 3D) and relapse (Fig. 3F). The time-dependent AUCs of the prognostic signature for HBV-associated HCC in the TCGA cohort were 0.824, 0.781, 0.781 and 0.796 for 1-year, 2-year, 3-year, and 5-year OS (Fig. 3E), and 0.650, 0.674, 0.674, and 0.654 for 1-year, 2-year, 3-year, and 5-year RFS, respectively (Fig. 3G). The nomogram of the FAM signature showed valuable and reliable probability for predicting the OS (Fig. 4) and RFS (Fig. 5) of HBV-associated HCC.

### Validation of the signature

**Fig. 6:**
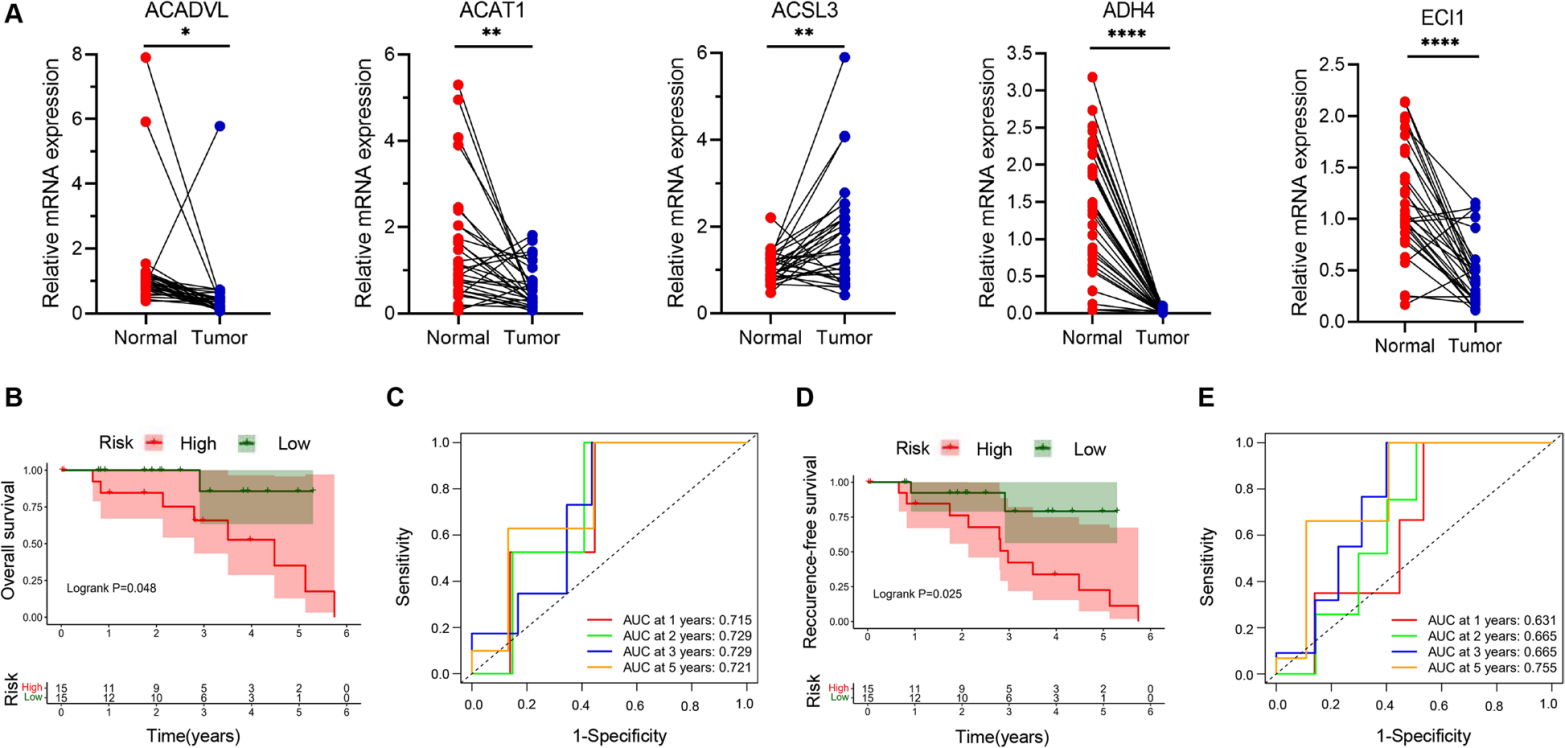
Performance of the prognostic signature for HBV-associated HCC in the validation cohort. **A** Relative mRNA expression of ACADVL, ACAT1, ACSL3, ADH4 and ECI1 in 30 HBV-associated HCC with paired normal tissue; **B** Kaplan-Meier curves for the OS; **C** Time-dependent ROC curves for predicting OS; **D** Kaplan-Meier curves for the RFS; **E** Time-dependent ROC curves for predicting RFS

**Fig. 7:**
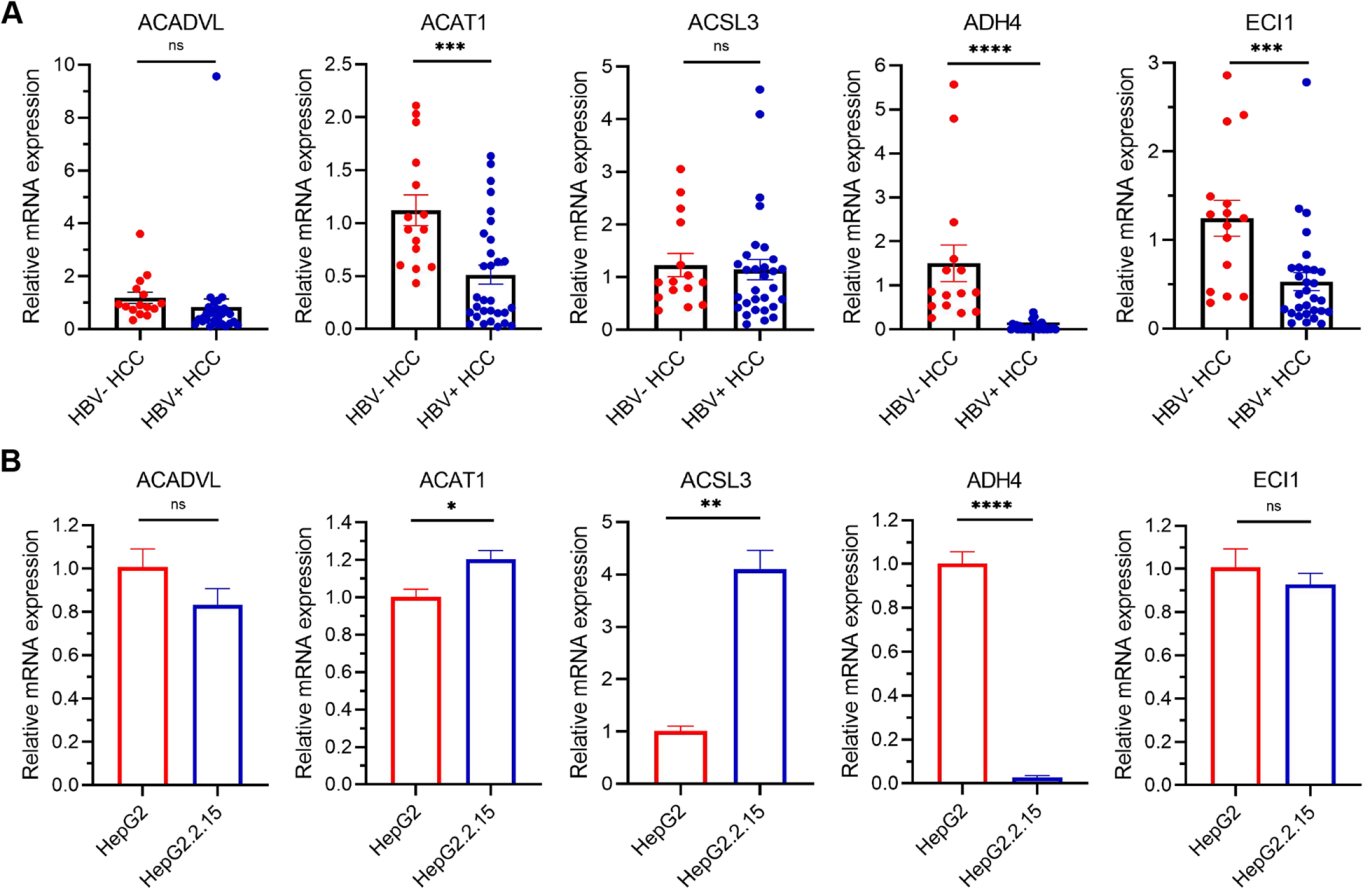
Differential expression of the prognostic signature genes between HBV-positive and -negative HCC patients and cell lines. **A** Relative mRNA expression between 30 HBV-positive HCC and 15 HBV-negative HCC patients; **B** Relative mRNA expression between HBV-positive and -negative HCC cell lines

In the cohort of the First Affiliated Hospital of Chongqing Medical University, 9 of 30 (30.00%) HBV-associated HCC patients died and 13 of 30 (43.33%) relapsed during the follow-up period. Firstly, expression at mRNA level of the five-gene signature showed that there was a significant difference between HBV-associated HCC tumor tissues and paired adjacent normal tissues (Fig. 6A). Likewise, Kaplan-Meier analysis showed that patients in the high-risk group had shorter OS (Fig. 6B) and RFS (Fig. 6D) time and were less likely to die and relapse. The time-dependent AUCs of the prognostic signature for HBV-associated HCC in the cohort of the First Affiliated Hospital of Chongqing Medical University were 0.715, 0.729, 0.729 and 0.721 for 1-year, 2-year, 3-year, and 5-year OS (Fig. 6C), 0.631, 0.665, 0.665 and 0.755 for 1-year, 2-year, 3-year, and 5-year RFS (Fig. 6E), respectively. To identify HBV-associated genes, mRNA expression level of the five-gene signature was assessed between 30 HBV-positive and 15 HBV-negative HCC tumor tissue, and in parental HepG2 cells compared with the daughter HepG2.2.15 cells containing two head-to-tail dimers of HBV genomic DNA (Sells et al., PNAS,1987). As a result, ACAT1, ADH4 and ECI1 were even more down-regulated in HBV-positive HCC tumor tissue (Fig. 7A), and ACAT1, ADH4 and ACSL3 were down-regulated in HepG2.2.15 to a higher degree (Fig. 7B). Taken together, ADH4 was simultaneously down-regulated in both HBV-positive HCC tumor tissue and HBV-positive HCC cell line, indicating that ADH4 might be a special biomarker for HBV-associated HCC.

### Immune cell infiltration

**Fig. 8:**
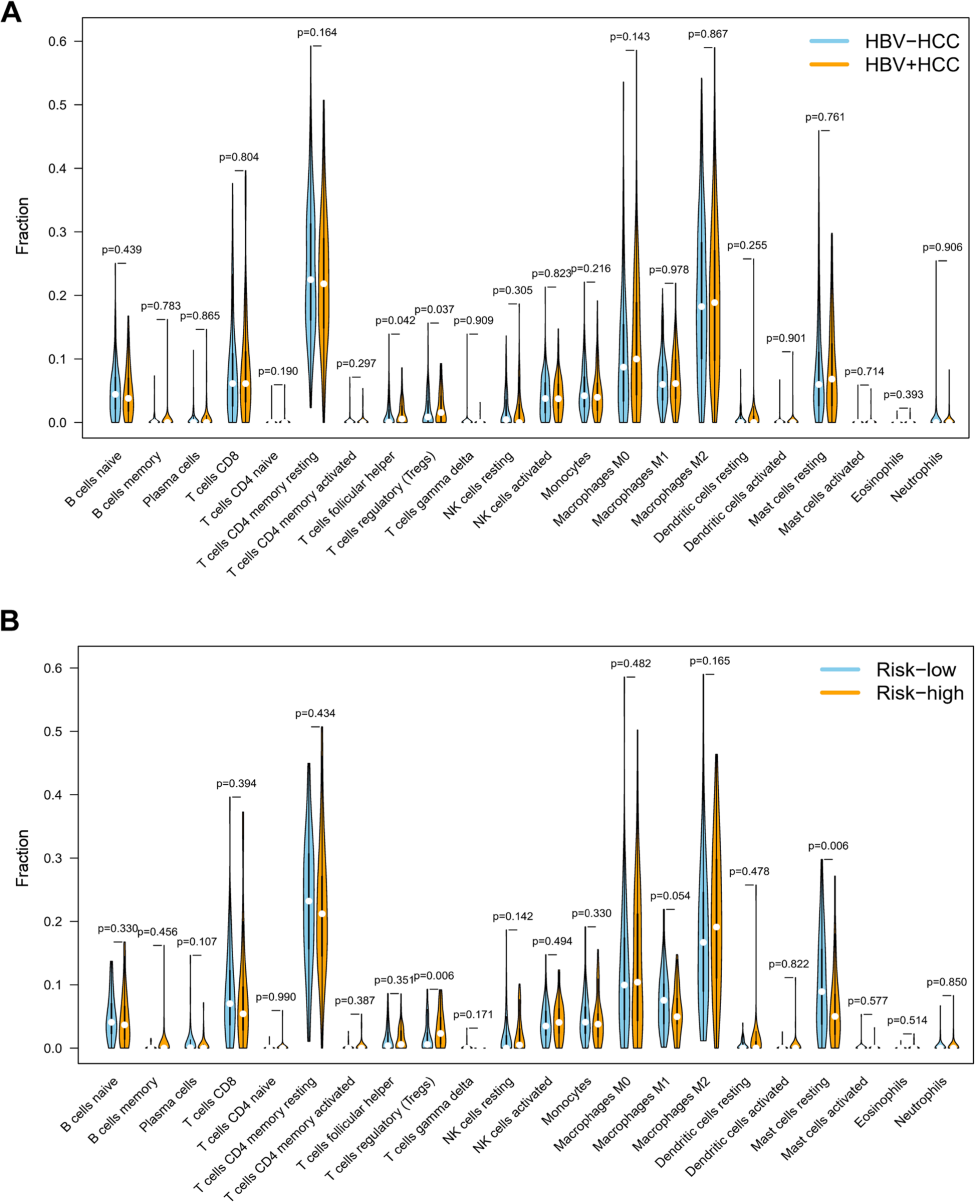
Differential analysis of immune infiltration. **A** Immune infiltration in HBV-positive (*n* = 117) and -negative (*n* = 254) HCC; **B** Immune infiltration between high-risk and low-risk group identified by the prognostic signature in HBV-positive HCC

**Fig. 9:**
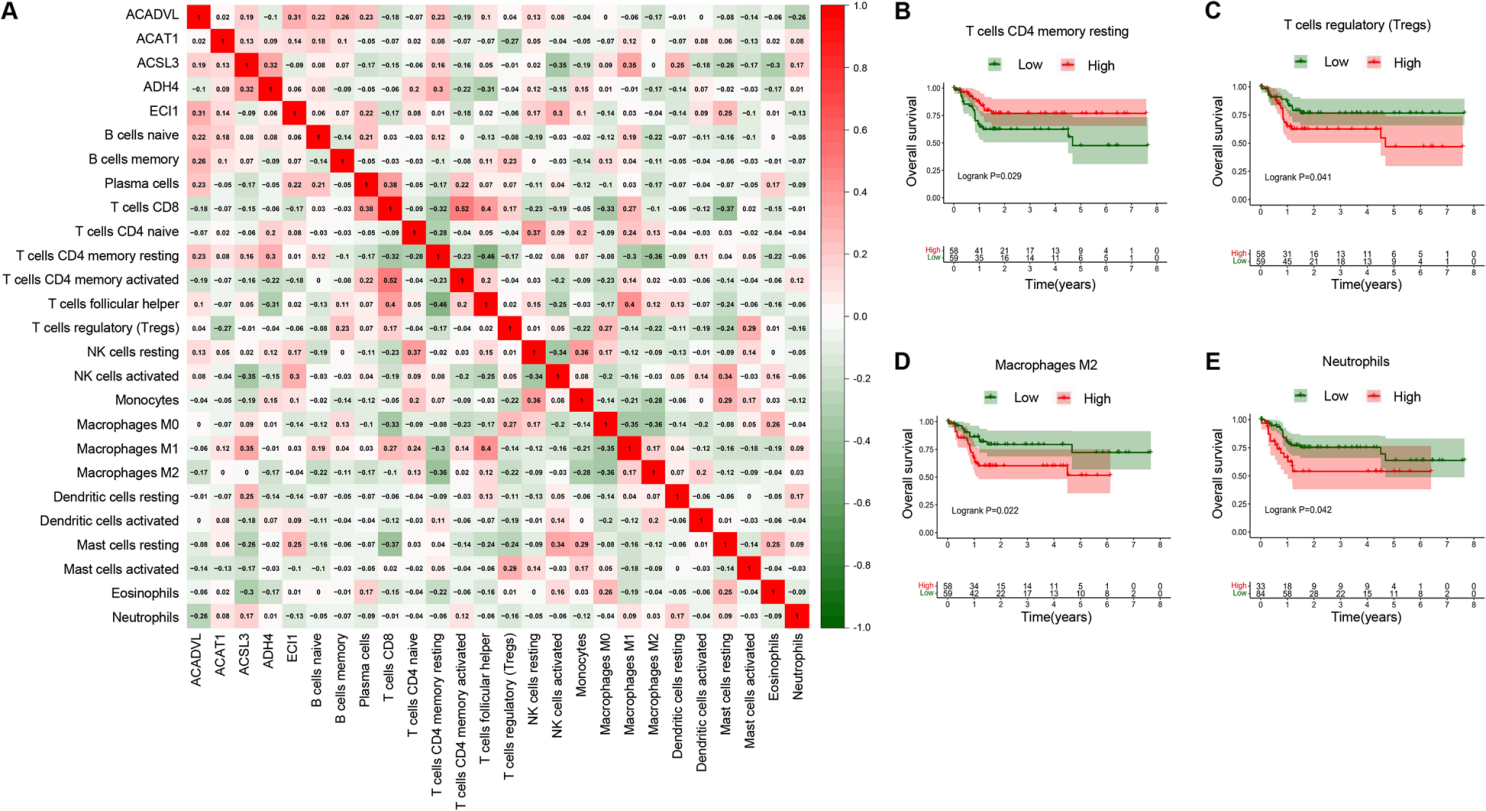
**A** Correlation analysis of 5 prognostic signature genes and 22 immune cells; Kaplan-Meier curves of four immune cells associated with overall survival in HBV-associated HCC: **B** T cells CD4 memory resting; **C** T cells regulatory (Tregs); **D** Macrophages M2; **E** Neutrophils

The proportion of 22 immune cells in groups of 117 HBV-positive and 254 HBV-negative HCC was evaluated by CIBERSORT (Fig. S3A and S3B). The proportion of 2 immune cells, T cells follicular helper and T cells regulatory (Tregs), showed significant differences between HBV-positive and HBV-negative HCC (Fig. 8A). According to the median RS, the proportion of Tregs and Mast cells resting were significantly differently infiltrated (Fig. 8B). HBV-positive HCC and high-risk group simultaneously had a higher ratio of Tregs, also suggesting that the identified FAM signature might be strongly correlated with HBV-associated HCC. The expression correlation between 5 FAM signature genes and 22 immune cells was shown in Fig. 9A. Through Kaplan-Meier analysis, it was found that the higher proportion of T cells CD4 memory resting, and the lower proportion of Tregs, Macrophages M2 and Neutrophils had a more favorable prognosis (Fig. 9B-E).

## Discussion

Hepatocellular carcinoma mainly develops within an established background of chronic liver inflammation caused by hepatitis B virus infection, hepatitis C virus (HCV) infection, uses of alcohol and non-alcoholic fatty liver disease (NAFLD) [4, 22]. HBV infection is the leading cause in eastern Asia and Africa, where the majority of HCC cases arises [4]. Persistent infection caused liver damage leads to metabolic disorders, especially the dysfuction of fatty acid metabolism. However, study investigating the linkage of fatty acid metabolism with tumor microenvironment in HBV-associated HCC, and its therapeutic strategies, remains largely unknown.

There are a variety clinical routine indicators available to guide the prognosis of HBV-associated HCC patients, which may offer benefits but also have limitations. It is well known that chronic hepatitis B (CHB) can progress to cirrhosis and eventually HCC. Host extrachromosomal covalently closed circular DNA (cccDNA, HBV’s stable extrachromosomal transcription template) interacts with the viral genome in HBV-infected cells. HBV infection cannot be fully eliminated from liver cells. The aim of treatment is to attain a “functional cure,” which involves eliminating HBsAg and reducing the number of cccDNA. Interferons (IFNs) injections or antiviral nucleos(t)ide analogues (NAs) are utilized to treat CHB infection. NAs greatly impede HBV DNA replication, resulting in difficulty detecting fluctuations in blood HBV DNA levels throughout treatment. However, serum Hepatitis B core-related antigen (HBcrAg) reflects the cccDNA amount and transcriptional activity in hepatocytes, making it a valuable indicator for monitoring patients with CHB receiving NAs therapy. Extensive evaluation has shown the importance of HBcrAg testing as a reliable, low-cost, and easy-to-use tool in managing CHB patients [23]. In patients with chronic hepatitis B, the serum HBcrAg level has correlations not only with serum HBV DNA level but also with intrahepatic HBV DNA and pre-genomic RNA, as well as intrahepatic cccDNA level and its transcriptional activity [24]. Therefore, long-term serum HBcrAg level monitoring is imperative. Preoperatively, alpha-fetoprotein (AFP) can serve as a prognostic indicator for postoperative outcomes in HCC patients with HBV infection history, and a high preoperative AFP level is an independent risk factor [25]. Based on its origin and affinity with lectins, AFP can be categorised into three heterogeneous plastid types: AFP-L1, AFP-L2 and AFP-L33. The ratio of AFP-L3 to AFP increases with the malignancy of the tumour. Research involving a meta-analysis of 15 studies on AFP-L3% and both overall and relapse-free survival rates in HCC patients [26] indicates that those with higher AFP-L3% had poorer overall survival and relapse-free survival. Further subgroup analysis has indicated that AFP-L3% is a potential prognostic indicator for HCC patients with HBV and HCV infection backgrounds undergoing diverse treatment methods. There are additional indicators that may anticipate the postoperative survival of HCC patients. These comprise blood cell indicators like red blood cell count and lymphocyte to monocyte ratio, liver function indicators including prealbumin (PA) and international normalized ratio (INR), as well as biochemical indicators such as alkaline phosphatase (ALP) and gamma-glutamyltransferase (GGT). Despite routine clinical tests providing much of the data for assessing postoperative survival in HCC patients, accurately predicting individual outcomes remains a clinical challenge. Gene signatures, however, have emerged as a valuable complement to traditional indicators, improving the accuracy of prognosis. Many bioinformatics analyses of extensive microarray and high-throughput sequencing data obtained from public databases are conducted to investigate molecular biological mechanisms and discover probable molecular markers that aid in the diagnosis and prognosis of various diseases.

In the present study, GSEA analysis demonstrated that FAM pathway was significantly downregulated in tumor tissue of HBV-associated HCC. There were 42 FAM genes in such pathway, 5 of them were involved in both fatty acid degradation and biosynthesis (ACSL1, ACSL3, ACSL4, ACSL5, ACSL6), and the rest of them were involved in fatty acid degradation. Except for ACSL3 and ACSL4, FAM genes were either downregulated in tumor tissue or found no significant difference, indicating that the function of fatty acid degradation was impaired in tumor of HBV-associated HCC.

Among 42 FAM genes, 12 of them were found to be associated with the prognosis of HBV-associated HCC patients, implying that FAM pathway played an important role in the development of HBV-associated HCC. A prognostic signature, consisting of five genes (ACADVL, ACAT1, ACSL3, ADH4 and ECI1), was established based on 42 FAM genes, which showed good performance for the prognosis of HBV-associated HCC both in the TCGA cohort and the validation cohort. According to our established prognostic signature, ACAT1, ACSL3 and ADH4 were able to predict the prognosis. In particular, ACSL3 (Acyl-CoA Synthetase Long Chain Family Member 3) converts free long-chain fatty acids into fatty acyl-CoA esters, and thereby play a dual role of lipid biosynthesis and fatty acid degradation [27]. Ndiaye H et al. [28] performed immunohistochemical staining confirming that the expression of ACSL3 was increased in HCC compared with normal liver. Few researches study on the effect of ACSL3 on liver disease. However, ACSL3 was reported as unfavorable prognostic marker in many other disease. Klasson TD et al. [29] reported that ACSL3 not only regulated the accumulation of lipid droplets in clear cell renal cell carcinoma but also modulated ferroptosis sensitivity in a manner dependent on the composition of exogenous fatty acids. Fernández LP et al. [30] found that ACSL3 worked as an risk factor in non-small cell lung cancer, and the overexpression of ACSL3 increased cell proliferation, migration, and invasion, altering metabolic properties of lung cancer cells. ACAT1 (Acetyl-CoA Acetyltransferase 1) is one of the enzymes that catalyzes the last step of the mitochondrial beta-oxidation pathway, an aerobic process breaking down fatty acids into acetyl-CoA [31]. Gu L et al. [32] demonstrated that ACAT1 depletion repressed tumor progression and combination of ACAT1 inhibitor with sorafenib retarded HCC development to a great extent in mice. ADH4 (Alcohol dehydrogenase 4) was reported to catalyze the NAD-dependent oxidation of either all-trans-retinol or 9-cis-retinol [33], and oxidize long chain omega-hydroxy fatty acids, such as 20-HETE [34]. Previous studies found that the expression of ADH4 was markedly reduced in HCC tumor tissues and identified as significant prognostic biomarker in HCC [20, 35, 36]. ACADVL (Acyl-CoA Dehydrogenase Very Long Chain) is targeted to the inner mitochondrial membrane where it catalyzes the first step of mitochondrial fatty acid beta-oxidation, an aerobic process breaking down fatty acids into acetyl-CoA and allowing the production of energy from fats [37, 38]. Zhu QW et al. [39] found that VLCAD, another name for ACADVL, inhibited the proliferation and invasion of hepatocellular cancer cells through regulating PI3K/AKT axis. ECI1 (Enoyl-CoA Delta Isomerase 1), also known as DCI, is a key mitochondrial enzyme involved in beta-oxidation of unsaturated fatty acids, catalyzing the transformation of 3-cis and 3-trans-enoyl-CoA esters arising during the stepwise degradation of cis-, mono-, and polyunsaturated fatty acids to the 2-trans-enoyl-CoA intermediates [40]. Rasmussen AL et al. [41] identified that DCI was required for hepatitis C virus replication and likely pathogenesis. However, few researches have investigated the relationship between those FAM genes and HBV-associated HCC. There seems to be much prospect of this exploration.

Moreover, immune infiltration analysis demonstrated that the high-risk group of HBV-associated HCC identified by the prognostic signature had a higher ratio of Tregs (regulatory T cells), which exerts immunosuppressive effect in TME. It has been reported that HBV infection facilitates the recruitment and accumulation of massive numbers of Tregs into the TME, which is in accordance with our findings, impeding effective antitumor responses and contributing to poor prognosis [5]. Those results suggest that targeting FAM could be a potential strategy to block the accumulation of Tregs into the TME of HBV-associated HCC.

Taken together, ADH4 was simultaneously down-regulated in both HBV-positive HCC tumor tissue and HBV-positive HCC cell line, indicating that ADH4 might be a special biomarker for HBV-associated HCC. Thus, targeting ADH4 to regulate fatty acid metabolism may be a potential strategy to block the accumulation of Tregs into the TME of HBV-associated HCC.

Compared with previous studies, the present study has several strengths. We used GSEA analysis to identify the performance of the whole FAM pathway - fatty acid degradation to be exact - rather than a few FAM-related genes. Based on FAM genes, a prognostic signature was established and showed effective discrimination and prediction for the OS and RFS of HBV-associated HCC not only in the TCGA cohort but also in the validation cohort. More importantly, the signature was associated with the infiltration of Tregs in TME, suggesting a potential strategy targeting FAM to block the accumulation of Tregs into the TME of HBV-associated HCC. Inevitably, there exists several limitations. These results were only based on the expression at mRNA level, and it will be more convincing if clinical fatty acid data is included. Finally, to confirm the evidence we found, further in-depth study and a larger cohort are needed to be explored.

## Conclusions

In conclusion, these findings suggest that there is a strong connection between fatty acid metabolism - fatty acid degradation to be exact - and HBV-associated HCC, indicating a potential therapeutic strategy for targeting fatty acid metabolism to block the accumulation of Tregs into the tumor microenvironment of HBV-associated HCC.

### Supplementary Information

**Additional file 1.**

**Additional file 2.**

**Additional file 3.**

## Data Availability

The datasets generated and analyzed during the current study are available in the Gene Expression Omnibus database (https://www.ncbi.nlm.nih.gov/geo/query/acc.cgi?acc=GSE94660 and https://www.ncbi.nlm.nih.gov/geo/query/acc.cgi?acc=GSE121248) and The Cancer Genome Atlas (TCGA) databases (https://portal.gdc.cancer.gov/) and are available from the corresponding author on reasonable request.

## Abbreviations

HCC: hepatocellular carcinoma
HBV: hepatitis-B virus
FAM: fatty acid metabolism
FA: fatty acid
GEO: Gene Expression Omnibus
TCGA: The Cancer Genome Atlas
GSEA: Gene Set Enrichment Analysis
KEGG: Kyoto Encyclopedia of Genes and Genomes
OS: overall survival
RFS: recurrence-free survival
ROC: receiver operating characteristic
AUC: area under the ROC curve
ACADVL: Acyl-CoA Dehydrogenase Very Long Chain
ACAT1: Acetyl-CoA Acetyltransferase 1
ACSL3: Acyl-CoA Synthetase Long Chain Family Member 3
ADH4: Alcohol dehydrogenase 4
ECI1: Enoyl-CoA Delta Isomerase 1
TME: tumor microenvironment
Tregs: regulatory T cells
