# Supplementary material for: Establishment of a prognostic signature based on fatty acid metabolism genes in HCC associated with hepatitis B
Source: BMC Gastroenterol. 2023 Nov 13;23:390. doi: 10.1186/s12876-023-03026-5 (PMC10644542; doi:10.1186/s12876-023-03026-5)
Supplement: Supplementary file 1 — Additional file 1. [file 12876_2023_3026_MOESM1_ESM.pdf]

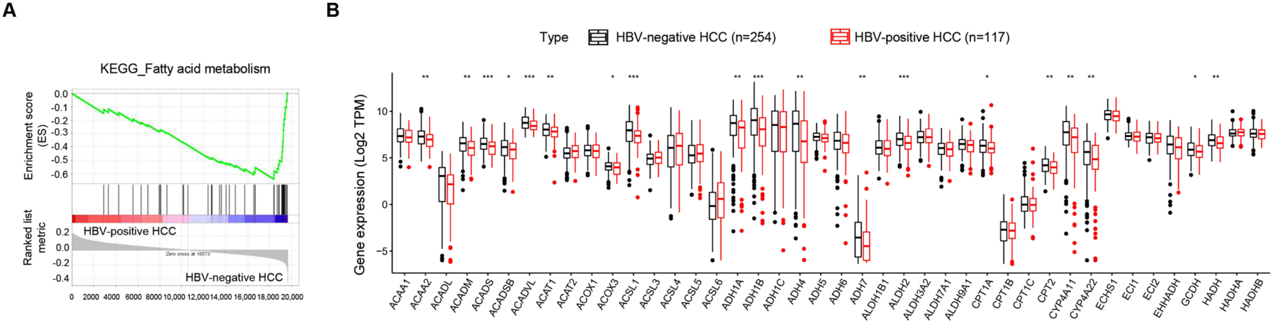

Figure S1. GSEA analysis of FAM pathway between HBV-positive and -negative HCC in the TCGA database. **A** GSEA results between HBV-positive and -negative HCC; **B** Specific FAM genes between HBV-positive and -negative HCC.
