# Supplementary material for: Establishment of a prognostic signature based on fatty acid metabolism genes in HCC associated with hepatitis B
Source: BMC Gastroenterol. 2023 Nov 13;23:390. doi: 10.1186/s12876-023-03026-5 (PMC10644542; doi:10.1186/s12876-023-03026-5)
Supplement: Supplementary file 2 — Additional file 2. [file 12876_2023_3026_MOESM2_ESM.pdf]

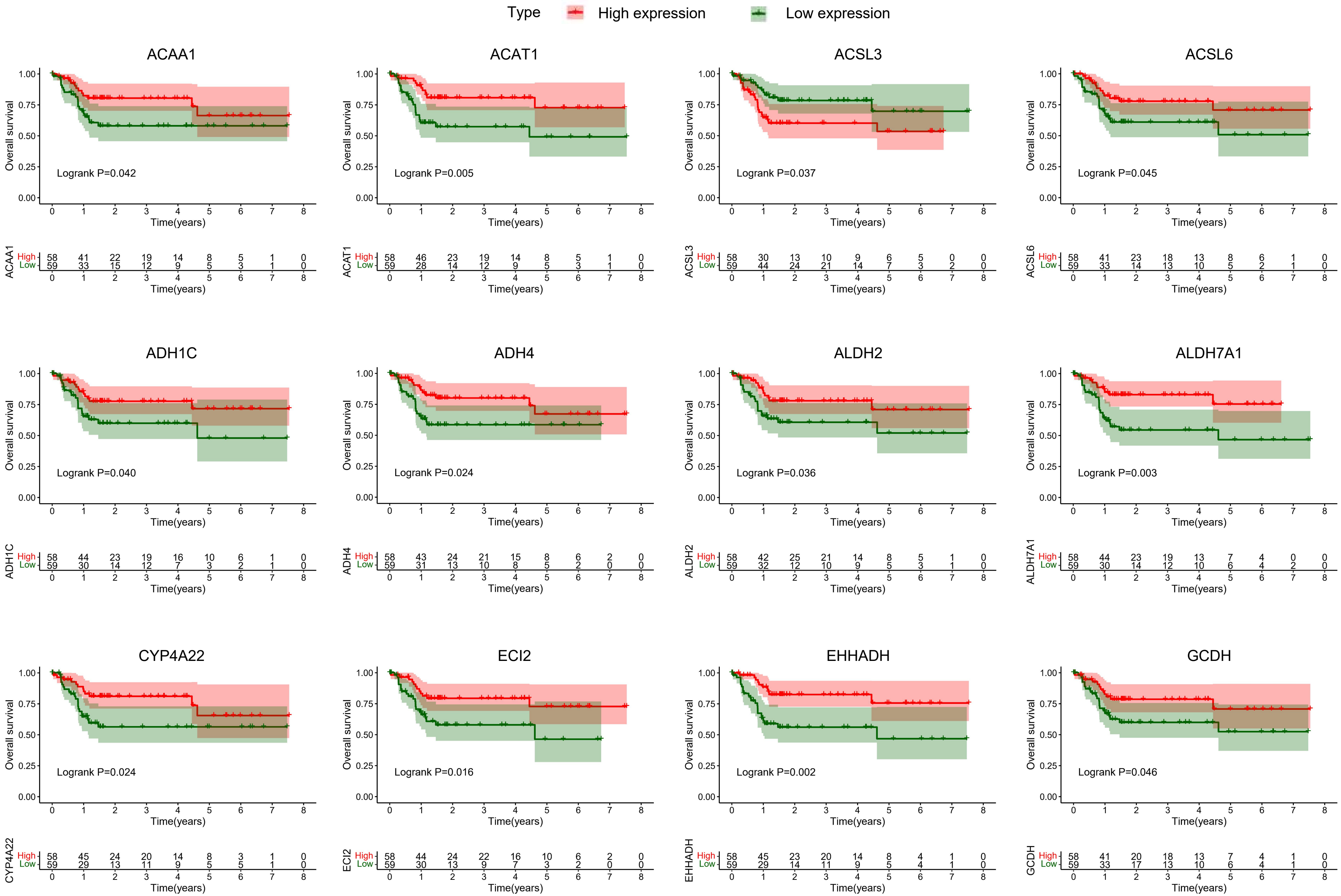

Figure S2. Kaplan-Meier curves of twelve genes associated with overall survival in HBV-associated HCC. The order of Kaplan-Meier curves of prognostic genes is as follows: ACAA1, ACAT1, ACSL3, ACSL6, ADH1C, ADH4, ALDH2, ALDH7A1, CYP4A22, ECI2, EHHADH, GCDH.
