# Supplementary material for: Establishment of a prognostic signature based on fatty acid metabolism genes in HCC associated with hepatitis B
Source: BMC Gastroenterol. 2023 Nov 13;23:390. doi: 10.1186/s12876-023-03026-5 (PMC10644542; doi:10.1186/s12876-023-03026-5)
Supplement: Supplementary file 3 — Additional file 3. [file 12876_2023_3026_MOESM3_ESM.pdf]

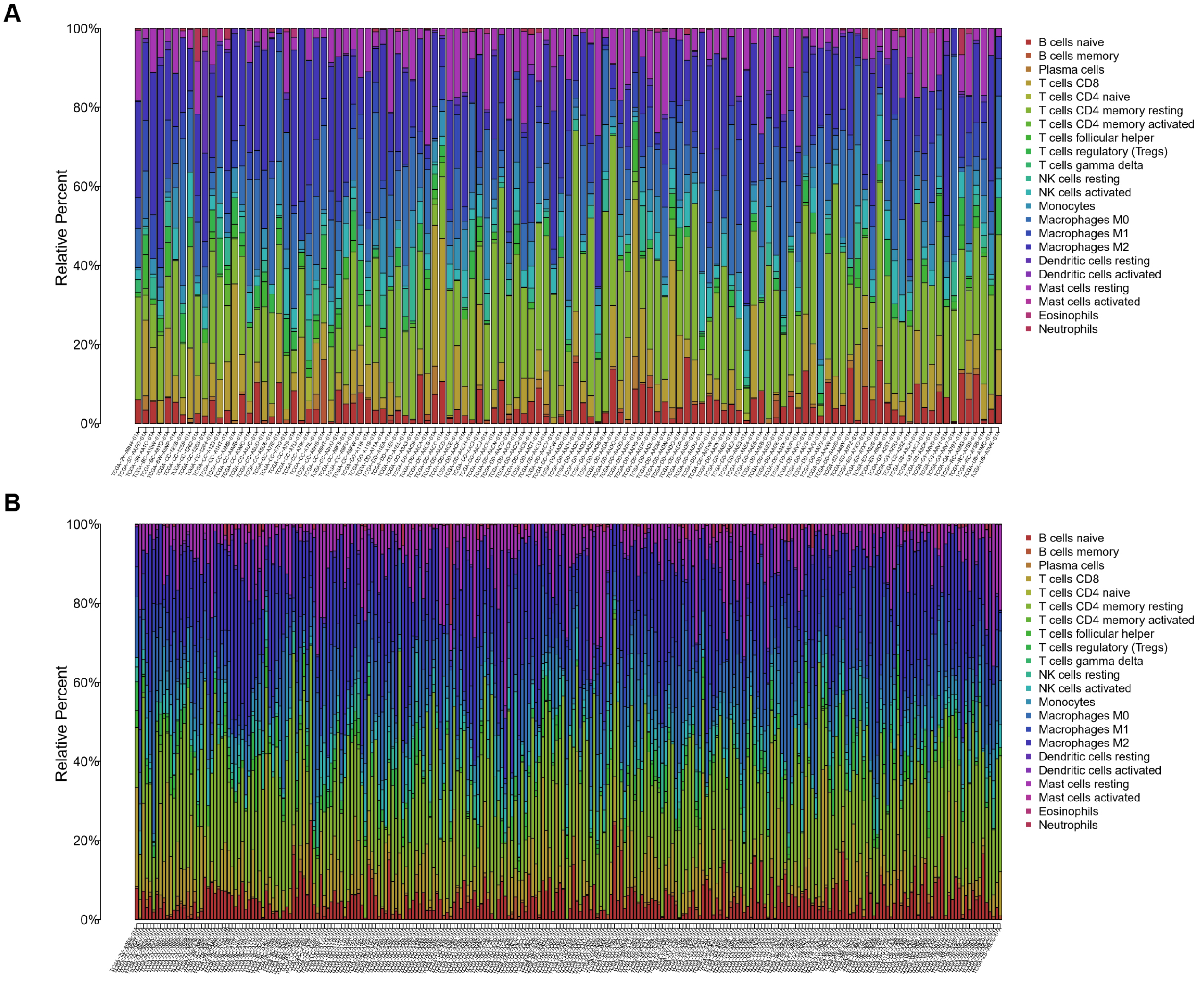

Figure S3. Immune infiltration analysis of HCC: **A** Immune infiltration of HBV-positive (n=117) HCC; **B** Immune infiltration of HBV-negative (n=254) HCC.
